# Extremely asymmetric ectasia: Tomographically unilateral keratoconus

**DOI:** 10.1111/aos.17456

**Published:** 2025-02-07

**Authors:** Hasan Shabani, Wishal D. Ramdas, Magda A. Meester‐Smoor, Dimitris Rizopoulos, Caroline C. W. Klaver, Bart T. H. van Dooren, Annette J. M. Geerards

**Affiliations:** ^1^ Department of Ophthalmology, Erasmus MC Erasmus University Medical Center Rotterdam The Netherlands; ^2^ Department of Epidemiology, Erasmus MC Erasmus University Medical Center Rotterdam The Netherlands; ^3^ Cornea Center Rotterdam Eye Hospital Rotterdam The Netherlands; ^4^ Department of Biostatistics, Erasmus MC Erasmus University Medical Center Rotterdam The Netherlands; ^5^ Department of Ophthalmology Radboud University Medical Center Nijmegen The Netherlands; ^6^ Amphia Hospital Breda The Netherlands

**Keywords:** forme fruste keratoconus, keratoconus, keratoconus aetiology, keratoconus epidemiology, keratoconus risk factors, Scheimpflug tomography, unilateral keratoconus, very asymmetric ectasia

## Abstract

**Purpose:**

To investigate the characteristics of apparently stable forms of tomographically unilateral keratoconus (KC).

**Methods:**

In this retrospective case–control study, strict unilaterality criteria were applied to select tomographically unilateral cases with ≥3 years of follow‐up. For comparison, a healthy cohort and two bilateral KC cohorts were matched to the tomographically unilateral cases. All patients were selected from The Rotterdam Eye Hospital, whereas healthy controls were selected from the population‐based Rotterdam Study. After cohort selection, several risk factors and 25 Pentacam features were assessed. Unaffected (i.e. tomographically non‐keratoconic) eyes from the tomographically unilateral cases were compared to matched healthy eyes, matched bilateral KC eyes and affected unilateral KC eyes. Furthermore, affected tomographically unilateral KC eyes were compared to matched bilateral KC eyes. Statistical analysis relied on Wilcoxon signed‐rank tests and conditional logistic regression.

**Results:**

From 1006 assessed cases, 18 (1.8%) tomographically unilateral cases were selected. Their median (interquartile range) follow‐up was 5.7 (4.3–8) years. Eczema and asthma were more prevalent among tomographically unilateral patients (28% each) compared to bilateral patients (6% and 9%) (*p* = 0.01 and *p* = 0.05, signed‐rank test). We could not detect meaningful Pentacam differences between unaffected unilateral eyes and matched healthy eyes. Expectedly, significant differences were detected between unaffected unilateral eyes and affected (bilateral or unilateral) eyes. Lastly, the ectatic features of affected unilateral eyes seemed comparable to their bilateral counterparts, but their high‐order aberrations were significantly lower.

**Conclusion:**

Our findings support the existence of tomographically unilateral KC. Understanding how tomographic unilaterality ensues may offer valuable insights into KC aetiology.

## 1 INTRODUCTION

Keratoconus (KC) is classically described as a bilateral asymmetric disorder. Despite being the most common form of corneal ectasia, the pathogenesis of KC remains poorly understood (Santodomingo‐Rubido et al., 2022). Many experts contend that true unilateral KC does not exist, a view that was endorsed by the last global consensus on KC and ectatic disorders (Gomes et al., 2015). Genuine unilateral KC cases, if existent, present a unique natural experiment, where the same genetic makeup gives rise to remarkably divergent phenotypes.

KC asymmetry exists on a spectrum, and very asymmetric KC forms where the fellow eye is not clearly abnormal are known to exist. Terms such as forme fruste KC, subclinical KC or very asymmetric KC are frequently used to refer to such cases (Henriquez et al., 2020; Nakao et al., 2021). Occasionally, the term unilateral KC might loosely be used to refer to these cases (Li et al., 2004). Semantics aside, several studies suggested that the less affected eyes in very asymmetric ectasia cases have some subtle ectatic changes and can therefore be distinguished from normal healthy eyes using advanced imaging and/or biomechanical assessment (Ambrósio et al., 2023; Bae et al., 2014; Fraenkel et al., 2021; Lu et al., 2023; Luz et al., 2016; Temstet et al., 2015; Thulasidas & Teotia, 2020; Tian et al., 2021; Yang, Qi, et al., 2024; Zhang et al., 2015).

A recent study suggested that some extreme forme fruste cases might actually be true unilateral KC cases (Guo et al., 2021). However, these extreme cases which border on true unilateral KC remain underexplored. A small case series based on Scheimpflug tomography featured five patients with what seems like genuine unilateral KC (Imbornoni et al., 2017). Similarly, two isolated case reports featured two patients with topographically, tomographically and biomechanically unilateral KC (Di Felici et al., 2023; Saad et al., 2022).

This study aimed to delve deeper into the apparently stable forms of tomographically unilateral KC. We assessed these cases using Pentacam Scheimpflug tomography (Oculus Optikgeräte GmbH, Wetzlar, Germany) and adequate follow‐up times in a relatively large cohort of KC patients. We also aimed to compare the identified tomographically unilateral cases to other healthy individuals and bilateral KC patients. Understanding these extremely asymmetric presentations may provide new insights into KC pathophysiology.

## 2 MATERIALS AND METHODS

This study is an observational, retrospective case–control study. Cases were recruited from the Rotterdam Eye Hospital, while healthy controls were recruited from The Rotterdam Study.

We assessed all KC patients who visited the Rotterdam Eye Hospital between June 2007 and September 2019 and had at least one reliable tomography scan (*n* = 1500). The diagnosis was ascertained based on the presence of characteristic clinical and Pentacam signs (Santodomingo‐Rubido et al., 2022).

The Rotterdam Study is a population‐based study conducted in Ommoord, The Netherlands. For the current analysis, we selected controls from the RS‐IV‐1 cohort (*n* = 3005) (Ikram et al., 2020). These participants were aged 40 years and over and underwent extensive ophthalmic examination, including Pentacam imaging.

### 2.1 Study groups

To address the research questions, selected Pentacam features (see further) of the tomographically unilateral KC cases were compared to those of healthy controls and those of bilateral KC patients. Five distinct groups were analysed in this study:

1. *UL fellow*: Unaffected fellow eyes of tomographically unilateral cases (*n* = 18).
2. *UL‐KC*: Keratoconic eyes of tomographically unilateral cases (*n* = 16, because two patients did not have reliable scans).
3. *Fellow‐matched KC*: Eyes of bilateral KC patients who were matched on age and sex to the UL Fellow group (*n* = 54).
4. *UL‐matched KC*: Eyes of bilateral KC patients who were matched on age and sex to the UL‐KC group (*n* = 48).
5. *Controls*: Controls without KC from the Rotterdam Study who were sex matched to the UL fellow group (*n* = 900).

Table S1 provides a quick summary of these groups. The names in bold will be used consistently from now on throughout this article to refer to each group. In the next subsections, we will explain how each group was selected.

### 2.2 Unilateral KC cases (UL fellow and UL‐KC)

A total of 1006 KC patients who had at least two reliable Pentacam scans, taken on two different occasions, were assessed for their eligibility as stable tomographically unilateral KC cases. To narrow down the search process, an initial list was compiled consisting of very asymmetric KC cases (*n* = 59). The Pentacam scans of these cases were exported in September 2022 and assessed by two corneal specialists (A.G. and B.v.D.) according to predetermined criteria. Table 1 lists the used criteria, which were chosen to ensure the exclusion of fellow eyes with tomographically detectable early ectasia (Cui et al., 2016; Heidari et al., 2021; Hersh et al., 2017; Kim et al., 2011; Li et al., 2004; Rabinowitz & Rasheed, 1999; Shetty et al., 2017; Wagner et al., 2007; Wittig‐Silva et al., 2014). A case was only included if the two specialists agreed that the eligibility criteria were satisfactorily met.

**TABLE 1:** Definition of stable tomographically unilateral keratoconus.

One eye manifests clinical keratoconus, while the fellow eye fulfils all of the following criteria: A clinical and Pentacam follow‐up of at least 36 months. No biomicroscopic sign(s) typical of keratoconus (Fleischer ring, scarring and Vocht's striae) present during the last exam (Wagner et al., 2007). No AB/SRAX pattern on the axial curvature map of the last good Pentacam scan (Li et al., 2004). Normal final D‐index (BAD‐D) of <1.6 in the last good Pentacam scan (Shetty et al., 2017). Normal posterior elevation at the thinnest point of <13 μm in myopes and < 22 μm in hyperopes (Kim et al., 2011). Normal ART‐max of >340 μm in the last good Pentacam scan (Shetty et al., 2017). Normal thinnest pachymetry reading of >506.5 μm in the last good Pentacam scan (Cui et al., 2016). Normal KISA% reading of <60% in the last good Pentacam scan (Rabinowitz & Rasheed, 1999). Normal *K*_max_ reading of <47.2 dioptres in the last good Pentacam scan (Heidari et al., 2021). The change in *K*_max_ across follow‐up is <1 dioptres (Hersh et al., 2017; Wittig‐Silva et al., 2014).

*Note*: Curvature maps were jointly assessed by two corneal specialists. AB/SRAX, a topographic pattern characterized by the presence of an asymmetric bowtie with skewed radial axes. ART‐max, highest reading of Ambrosio's relational thickness; BAD‐D, the final D of the Belin–Ambrosio enhanced ectasia display; KISA%, a composite topographic index; *K*_max_, highest keratometry of the corneal front.

### 2.3 Bilateral KC cases (UL‐matched KC and fellow‐matched KC)

Two distinct groups of bilateral KC patients were selected from the Rotterdam Eye Hospital KC database. To be considered, patients needed to have bilateral KC, at least one reliable Pentacam scan and a completed KC questionnaire. In total, 658 patients were considered for the formation of the following two groups:

1. *The UL‐matched KC group*: Including bilateral KC patients matched for age and sex with the UL‐KC group. The objective of creating this group was to facilitate direct comparisons between the affected unilateral corneas, as presented in this article, and bilateral KC cases. Specifically, for each UL‐KC case, three bilateral patients were selected as matches.
2. *The fellow‐matched KC group*: Including bilateral KC patients matched for age and sex with the UL fellow group. The primary objective for this group was to facilitate comparing the presented healthy fellow corneas to typical KC eyes. For each fellow KC case, three bilateral patients were selected as matches.

When creating each bilateral KC group, one random eye was selected from each matched patient. Age matching was performed using a ±4 years criterion. Given this criterion, it was not feasible to match more than three bilateral patients to each tomographically unilateral patient. Crosslinking can alter corneal morphology and Pentacam readings. Therefore, readings from the last good Pentacam scan before corneal crosslinking or keratoplasty, if applicable, were used. Age was calculated based on the date of the selected relevant scan. This approach led to significant age distribution disparities between the UL‐KC group, many of whom had been operated on before, and the healthy UL‐fellow group. These disparities necessitated the creation of two separate bilateral KC reference groups.

### 2.4 Healthy controls

A total of 2201 RS‐IV‐1 participants had at least one reliable Pentacam scan. Eyes with a history of corneal pathologies, interventions or contact lens usage were excluded. Only individuals with at least one healthy cornea were considered eligible to be selected as controls (*n* = 2063). For each tomographically unilateral case, 50 random controls were sex matched (total *n* = 900). A random eye was selected from each matched control for the statistical analysis. Age matching was not feasible given the differences in age distribution between the two cohorts.

### 2.5 Scheimpflug tomography scans

The Pentacam utilizes a primary Scheimpflug camera that captures images in three planes. During each scan, data from as many as 138 000 independent elevation points are collected and used to generate a three‐dimensional model of the anterior eye segment (Oculus, 2023). After the scan, many parameters, indices and maps are generated by the Pentacam software. Only scans that had an ‘OK’ quality score as given by Pentacam were considered reliable and used in this study.

A total of 25 Pentacam variables were analysed in the comparative analysis. Sixteen of these features are highly relevant for detecting and defining (early) ectasia, and nine represent key wavefront aberrations that usually accompany ectasia.

### 2.6 Medical history

Information regarding ocular inflammation, allergies, asthma, dust mite allergy, hay fever, eczema and food allergy were extracted from the relevant records. At The Rotterdam Eye Hospital, patients were routinely assessed for ocular inflammation during clinical evaluations and asked about the other risk factors using a standardized ‘yes/no’ format. For Rotterdam Study controls, dust mite allergy and hay fever are collected in a similar ‘yes/no’ format during regular follow‐up interviews, whereas asthma is established based on a careful assessment of medical history as described elsewhere (de Roos et al., 2018). Unfortunately, data about eczema, food allergy and ocular inflammation were not available for the Rotterdam Study controls.

### 2.7 Statistical analysis

The previously mentioned selected Pentacam features of UL‐KC eyes and UL‐fellow eyes were compared using the Wilcoxon signed‐rank test. Additionally, conditional logistic regression models were used to compare the same Pentacam features between the UL‐KC group and the corresponding matched bilateral keratoconic eyes group (UL‐matched KC). The same modelling approach was used to compare UL‐fellow eyes with the fellow‐matched KC eyes. Moreover, conditional logistic regression was performed with and without age correction to compare the UL‐fellow group with the healthy control group. Some of the analysed Pentacam variables were transformed (multiplied by 100) to obtain more meaningful confidence intervals and resolve numerical problems (Hosmer et al., 2013). Given that a total of 25 variables were tested, results were considered significant at or below a Bonferroni‐adjusted significance level of 0.002.

Nested linear mixed models were used to compare the follow‐up course of the affected UL‐KC eyes and the healthy UL‐fellow eyes. Eye status (affected or not) and follow‐up length were included as fixed effects. The models also incorporated two nested random‐effect levels: one representing individual variability and another capturing variation within each individual. Random intercepts were included at both levels for model fitting. The outcome parameters analysed are the three tomographic parameters of the Pentacam's progression display. These parameters are the ARC (anterior radius of curvature), PRC (posterior radius of curvature) and the thinnest pachymetry. The analysed period was limited to the first 60 months of follow‐up counted from the date of their first Pentacam scan. This choice was made to prevent cases with long follow‐ups from skewing the analysis.

### 2.8 Ethical considerations

The study was conducted according to the tenets of the Declaration of Helsinki. The Rotterdam Study has been approved by the Medical Ethics Committee of the Erasmus MC. All Rotterdam Study participants provided written informed consent to participate in the study and to have their information obtained from treating physicians. The part of the study which involves patients from the Rotterdam Eye Hospital was done in harmony with the current Dutch laws that exempt retrospective research on patient files from obtaining a medical ethical review.

## 3 RESULTS

### 3.1 Selected unilateral cases

Among 1006 screened KC patients, a total of 18 (1.8%) cases met the eligibility criteria and were considered stable cases of tomographically unilateral KC according to our definition. The mean (range) follow‐up of this cohort was 6.6 (3.8–12.6) years, and the median (interquartile range) follow‐up was 5.7 (4.3–8) years. Their mean (range) and median (IQR) ages at the last follow‐up were 34.9 (23–70.5) and 31 (26.3–41.3) years, respectively. The mean age at diagnosis of the tomographically unilateral cases was 25.2 years (range: 12.8–52.1) based on the 16 cases where the age at diagnosis could be determined.

### 3.2 Study groups' characteristics

**FIGURE 1:**
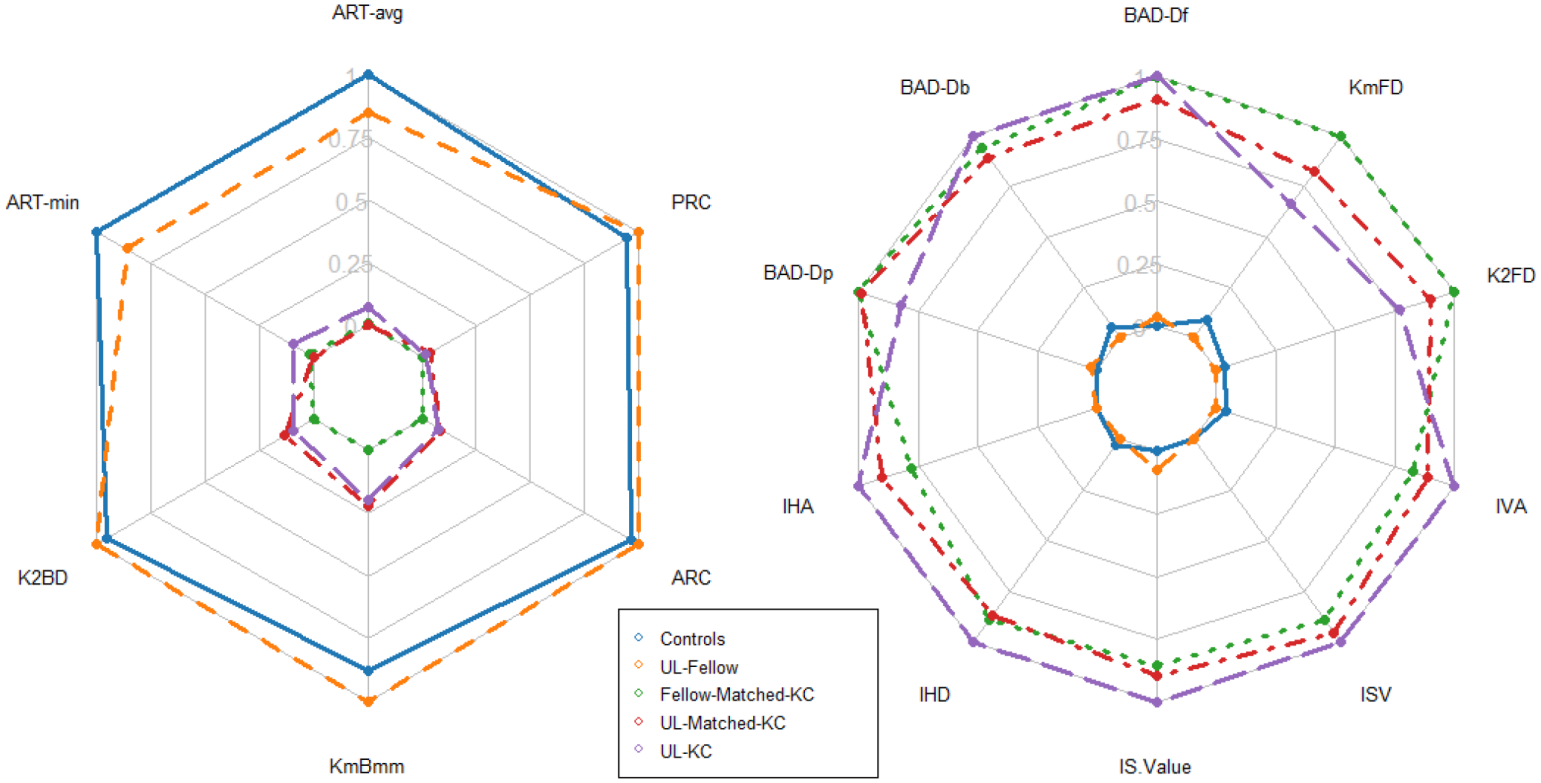
Spider plots showing the means of key Pentacam ectasia variables across study groups. Min–max scaling was applied to transform the means. The left plot shows variables that decrease with ectasia while the right plot shows variables that increase with ectasia. ARC, anterior radius of curvature; ART‐avg, average Ambrosio relational thickness; ART‐min, minimum Ambrosio relational thickness; BAD‐Db, standardized back elevation difference between the best‐fit sphere and the enhanced reference surface; BAD‐Df, standardized front elevation difference between the best‐fit sphere and the enhanced reference surface; BAD‐DP, standardized average pachymetric progression index; IHA, index of height asymmetry (microns); IHD, index of height difference (microns); ISV, index of surface variance; IS value, inferior–superior value (dioptres); IVA, index of vertical asymmetry (millimetres); K2BD, mean keratometry in the steepest meridian of the cornea's back (dioptres); K2FD, mean keratometry in the steepest meridian of the cornea's front (dioptres); KmBmm, mean back surface keratometry (millimetres); KmFD, mean front surface keratometry; PRC, posterior radius of curvature.

Table 2 summarizes the general characteristics of the different study groups while Figure 1 and Table S2 show the distribution of their Pentacam features. Among the presented unilateral patients, 5 (28%) had asthma. This is significantly more than the proportion with asthma in the fellow‐matched KC group (9%), and in the healthy controls group (9%). Eczema was also significantly more common in unilateral patients (28%) than their bilateral counterparts (6%). Other relevant conditions, such as dust mite allergy and hay fever, seemed more common in unilateral patients, but the differences did not reach statistical significance.

**TABLE 2:** General characteristics of study participants presented as *N* (%) unless otherwise indicated.

	UL‐fellow (*N* = 18)	Fellow‐matched KC (*N* = 54)	UL‐matched KC (*N* = 48)	Controls (*N* = 900)	*p* Value (UL‐fellow vs. fellow‐matched KC)	*p* Value (UL‐fellow vs. controls)
Age,^a^ years, Median (IQR)	31 (26.3–41.3)	31.9 (28.3–39.8)	30.7 (26.5–40)	53.6 (48.4–58.4)	^b^	<0.01*
Female	7 (39%)	21 (39%)	21 (44%)	350 (39%)	^b^	^b^
OD	9 (50%)	27 (50%)	23 (48%)	440 (49%)	1	0.93
Ocular Inf.	6 (33%)	29 (54%)	22 (46%)	NA	0.142	^c^
Allergy (any)	9 (50%)	29 (54%)	24 (50%)	NA	0.783	^c^
Asthma	5 (28%)	5 (9%)	6 (13%)	87 (10%)	<0.05*	0.02*
DMA	4 (22%)	4 (7%)	8 (17%)	110 (12%)	0.097	0.22
Hay Fever	5 (28%)	10 (19%)	11 (23%)	156 (17%)	0.396	0.27
Eczema	5 (28%)	3 (6%)	4 (8%)	NA	0.018*	^c^
Food Allergy	3 (17%)	5 (9%)	2 (4%)	NA	0.396	^c^

*Note*: *p* Values were calculated using conditional logistic regression. Missing data points are indicated with NA. None of the other groups/variables in this study had any missing data points.

Abbreviations: DMA, dust mite allergy; Inf., inflammation; IQR, interquartile range.

^a^ Calculated at the last good Pentacam scan before corneal crosslinking or keratoplasty, if operated.

^b^ Variable used for matching.

^c^ Testing not feasible because of missingness.

* Statistically significant at a *p* value of 0.05.

### 3.3 Comparative analysis results

Table 3 compares the major Pentacam‐derived ectatic features among the different study groups. None of the compared ectasia variables showed significant differences when comparing unilateral keratoconic eyes (UL‐KC) to bilateral KC cases (UL‐matched KC). In contrast, the healthy UL‐fellow eyes showed some significant differences in key ectasia parameters such as the mean anterior and posterior keratometry when compared to the unilateral keratoconic (UL‐KC) or bilateral keratoconic (fellow‐matched KC) groups. No statistically significant differences emerged when comparing the ectatic features of UL‐fellow eyes to the sex‐matched Rotterdam Study controls (Table 3). This finding remained consistent even when age correction was applied (data not shown). Similar trends are observed in Table 4, which compares some of the most relevant wavefront aberrations between the studied groups. Notably, however, UL‐KC eyes had significantly lower RMS‐HOA (root mean square of high‐order aberrations) than UL‐matched KC eyes.

**TABLE 3:** Comparison of major ectatic parameters and indices between the studied groups.

Pentacam variable	UL‐KC vs. UL‐matched KC^a^ (16 vs. 48 eyes)		UL‐fellow vs. fellow‐matched KC^a^ (18 vs. 54 eyes)		UL‐KC vs. UL‐fellow^b^ (16 vs. 16 eyes)		UL‐fellow vs. controls^a^ (18 vs. 900 eyes)	
	*p*	OR (95%CI)	*p*	OR (95%CI)	*p*	*Z*	*p*	OR (95%CI)
BAD‐Df	0.565	1.033 (0.925–1.152)	0.001*	0.370 (0.206–0.665)	<0.001*	−3.516	0.231	1.225 (0.879–1.706)
BAD‐Db	0.488	1.051 (0.912–1.211)	0.001*	0.225 (0.092–0.555)	<0.001*	−3.516	0.118	0.665 (0.398–1.109)
BAD‐Dp	0.362	0.939 (0.821–1.075)	0.002*	0.213 (0.080–0.564)	<0.001*	−3.516	0.363	1.241 (0.779–1.979)
IHA	0.739	1.004 (0.983–1.025)	0.003	0.855 (0.770–0.950)	<0.001*	−3.516	0.976	1.001 (0.924–1.085)
IHD^c^	0.415	1.043 (0.943–1.153)	0.004	0.162 (0.048–0.551)	<0.001*	−3.516	0.222	0.666 (0.346–1.279)
IS value	0.499	1.060 (0.896–1.254)	0.001*	0.223 (0.095–0.523)	<0.001*	−3.516	0.018	1.767 (1.102–2.834)
ISV	0.758	1.003 (0.985–1.021)	0.002	0.825 (0.729–0.934)	<0.001*	−3.517	0.963	1.001 (0.940–1.067)
IVA^c^	0.454	1.005 (0.992–1.019)	0.002*	0.804 (0.701–0.921)	<0.001*	−3.517	0.060	0.920 (0.844–1.004)
ART‐avg	0.340	1.003 (0.997–1.009)	0.001*	1.025 (1.010–1.040)	<0.001*	−3.516	0.048	0.995 (0.991–1.000)
ART‐min	0.390	1.001 (0.999–1.004)	<0.001*	1.010 (1.005–1.015)	<0.001*	−3.464	0.182	0.998 (0.996–1.001)
K2BD^c^	0.875	0.999 (0.992–1.007)	0.001*	1.032 (1.013–1.051)	<0.001*	−3.356	0.536	1.005 (0.988–1.023)
K2FD	0.505	0.940 (0.785–1.127)	<0.001*	0.423 (0.262–0.685)	<0.001*	−3.408	0.599	0.922 (0.681–1.248)
KmBmm^c^	0.928	1.000 (0.991–1.008)	0.002*	1.027 (1.010–1.045)	<0.001*	−3.331	0.126	1.015 (0.996–1.034)
KmFD	0.443	0.922 (0.750–1.134)	<0.001*	0.442 (0.281–0.697)	<0.001*	−3.517	0.243	0.826 (0.600–1.138)
ARC	0.930	0.999 (0.988–1.011)	0.003	1.097 (1.031–1.168)	<0.001*	−3.517	0.543	1.005 (0.988–1.022)
PRC^c^	0.857	0.999 (0.989–1.009)	0.003	1.080 (1.027–1.136)	<0.001*	−3.517	0.277	1.010 (0.992–1.028)

Abbreviations: ARC, anterior radius of curvature in the 3‐mm zone centred around the thinnest pachymetry; ART‐avg, average Ambrosio relational thickness; ART‐min, minimum Ambrosio relational thickness; BAD‐Db, standardized back elevation difference between the best‐fit sphere and the enhanced reference surface; BAD‐Df, standardized front elevation difference between the best‐fit sphere and the enhanced reference surface; BAD‐DP, standardized average pachymetric progression index; CI, confidence interval; IHA, index of height asymmetry (microns); IHD, index of height difference (microns); ISV, index of surface variance; IS value, inferior–superior value (dioptres); IVA, index of vertical asymmetry (millimetres); K2BD, mean keratometry in the steepest meridian of the cornea's back (dioptres); K2FD, mean keratometry in the steepest meridian of the cornea's front (dioptres); KmBmm, mean back surface keratometry (millimetres); KmFD, mean front surface keratometry (dioptres); OR, odds ratio; *p*, *p* value; PRC, posterior radius of curvature in the 3‐mm zone centred around the thinnest pachymetry; *Z*, *Z*‐value.

^a^ Conditional logistic regression.

^b^ Wilcoxon signed‐rank test.

^c^ These variables were transformed (multiplied by 100) to improve numerical stability.

* Statistically significant results at a Bonferroni‐corrected *p* value of 0.002.

**TABLE 4:** Comparison of corneal wavefront aberrations between the studied groups.

Pentacam variable	UL‐KC vs. UL‐matched KC^a^ (16 vs. 48 eyes)		UL‐fellow vs. Fellow‐matched KC^a^ (18 vs. 54 eyes)		UL‐KC vs. UL‐fellow^b^ (16 vs. 16 eyes)		UL‐fellow vs. controls^a^ (18 vs. 900 eyes)	
	*p*	OR (95%CI)	*p*	OR (95%CI)	*p*	*Z*	*p*	OR (95%CI)
RMS (HOA) CB^c^	0.001*	0.896 (0.839–0.957)	^d^	^d^	<0.001*	−3.517	<0.001^e,^, *	0.231 (0.135–0.396)
RMS (HOA) CF^c^	0.001*	0.982 (0.971–0.993)	0.151	0.804 (0.597–1.083)	<0.001*	−3.516	<0.001^e,^, *	0.739 (0.665–0.821)
RMS (HOA) C^c^	0.001*	0.977 (0.963–0.991)	^d^	^d^	<0.001*	−3.516	<0.001^e,^, *	0.711 (0.630–0.801)
Z(3,‐1) CB^c^	0.003	0.958 (0.931–0.985)	0.004	0.883 (0.811–0.960)	<0.001*	−3.517	0.116	1.054 (0.987–1.125)
Z(3,‐1) CF^c^	0.006	1.010 (1.003–1.017)	0.001*	1.042 (1.017–1.068)	<0.001*	−3.516	0.038	0.985 (0.971–0.999)
Z(3,‐1) C^c^	0.005	1.012 (1.004–1.020)	0.001*	1.047 (1.019–1.076)	<0.001*	−3.517	0.060	0.987 (0.973–1.001)
Z(3,1) CB^c^	0.293	1.013 (0.989–1.039)	0.543	1.007 (0.984–1.030)	0.605	−0.517	0.562	0.966 (0.859–1.086)
Z(3,1) CF^c^	0.494	0.998 (0.991–1.004)	0.289	0.996 (0.990–1.003)	0.215	−1.241	0.586	0.993 (0.966–1.019)
Z(3,1) C^c^	0.553	0.998 (0.990–1.005)	0.251	0.996 (0.988–1.003)	0.196	−1.293	0.546	0.992 (0.967–1.018)

Abbreviations: C, total cornea; CB, corneal back surface; CF, corneal front surface; CI, confidence interval; OR, odds ratio; *p*, *p* value; RMS (HOA), root mean square of high order aberrations (microns); *Z*, *Z*‐value; *Z*(3,1), horizontal coma (microns); *Z*(3,−1), vertical coma (microns).

^a^ Conditional logistic regression.

^b^ Wilcoxon signed rank test.

^c^ These variables were transformed (multiplied by 100) to improve numerical stability.

^d^ Lack of convergence due to (quasi) perfect separation between the analysed groups (i.e. the UL‐fellow group had much lower aberrations compared to the fellow‐matched KC group).

^e^ UL‐fellow eyes had less aberrations (i.e. they were even healthier) than the healthy controls.

* Statistically significant results at a Bonferroni‐corrected *p* value of 0.002.

Linear mixed models showed some notable differences between the follow‐up course of the healthy UL‐fellow eyes when compared to their keratoconic UL‐KC counterparts. The ARC progressed significantly faster in the UL‐KC group compared to UL‐fellow eyes (*β* = −0.019 mm/year; 95%CI: −0.002 to −0.036; *p* = 0.031). This was also the case for the PRC (*β* = −0.038 mm/year; 95%CI: −0.011 to −0.064; *p* = 0.005). For the thinnest pachymetry, the estimated difference was −1.551 microns/year (95%CI: −3.214 to 0.152), but this did not reach statistical significance (*p* = 0.071).

## 4 DISCUSSION

In this study, we present an intriguing case series of stable tomographically unilateral KC. The statistical comparative analysis showed that the UL‐fellow eyes did not have more ectasia compared to healthy control eyes from the Rotterdam Study based on their topographic, tomographic and wavefront aberrometry measurements. The analysis also confirmed that the UL‐fellow eyes had clearly different corneal features and follow‐up course when compared to the UL‐KC eyes.

### 4.1 Evolving perspectives on unilateral KC

Corneal imaging has developed rapidly in the last few decades. While older Placido‐based topographers can only capture the anterior corneal surface, Scheimpflug tomography allows for the assessment of both corneal surfaces simultaneously. Many believe that corneal tomography has advantages over topography in detecting early ectatic changes (Gomes et al., 2015; Wolf et al., 2009). Therefore, it was not possible to directly compare the findings of our study with some older reports about unilateral KC, which relied mainly on clinical examinations and topography to define unilaterality (Holland et al., 1997; Lee et al., 1995; Rabinowitz et al., 1993).

Using Scheimpflug tomography, one study featured five unilateral KC patients with progressive KC on one side and no significant Pentacam changes on the other (Imbornoni et al., 2017). The five fellow eyes had a mean age of 27 years and a mean follow‐up of 59 months. Our analysis shows comparable results in a larger and older group of patients with a longer follow‐up. Similarly, two recent case reports featured two adults aged 19 and 17 at first presentation with what seems like genuinely unilateral KC (Di Felici et al., 2023; Saad et al., 2022). The fellow eye in these two reports remained topographically, tomographically and biomechanically stable during a follow‐up of 14 years and 3 years respectively. These studies suggest that stable forms of tomographically unilateral KC are present in other contexts as well.

### 4.2 Healthy unilateral eyes versus healthy control eyes

Our analysis of 25 key Pentacam features failed to detect meaningful differences between UL‐fellow eyes and the healthy controls. Of note, the statistically significant differences seen in Table 4 between these two groups are due to UL‐fellow eyes being less aberrant than normal eyes (see Table S2). This is probably a result of our strict eligibility criteria which led to selecting UL‐fellow eyes that have less aberrations than average normal eyes.

### 4.3 Unilateral versus bilateral KC

Interestingly, UL‐KC eyes had significantly lower RMS‐HOA than the UL‐matched KC cases in our study, highlighting an interesting difference between the two groups. Our analysis also suggests that eczema and asthma might be more frequent in tomographically unilateral KC patients than their bilateral counterparts. Notably, the mean age at diagnosis of the tomographically unilateral cases (25 years) was close to the previously reported mean age of KC diagnosis in The Netherlands (28 years) (Godefrooij et al., 2017).

### 4.4 Eye rubbing and unilateral KC

Numerous studies have identified eye rubbing as a major risk factor for KC (Santodomingo‐Rubido et al., 2022). Other recent studies have also suggested that rubbing cessation interventions may slow or halt disease progression (Mazharian et al., 2023; Yang, Liu, et al., 2024). It seems therefore plausible to suspect that excessive eye rubbing on one side may play a role in the development of highly asymmetric ectasia. A recent case–control study concluded that asymmetric rubbing and incorrect sleeping positions are risk factors for unilateral and highly asymmetric KC (Mazharian et al., 2020). Unfortunately, data on eye rubbing and sleeping habits were unavailable in our cohort. Interestingly though, there was an equal number of right and left affected unilateral eyes among the presented cases, suggesting no clear lateralization in our cohort. The interplay of genetics, handedness, eye rubbing and biomechanics in the pathogenesis of unilateral KC warrants further investigation. Further studies incorporating these variables would help clarify their contributions to the development and progression of KC, particularly in unilateral cases.

### 4.5 Strengths and limitations

Our study presents the largest published cohort of stable unilateral KC cases with consistent, uneventful tomographic and clinical follow‐up. Nevertheless, recruiting more unilateral patients would have improved the power of our analysis. Unlike some other studies examining very asymmetric ectasia, we used very stringent criteria to ensure the exclusion of fellow eyes with tomographically detectable subclinical ectasia. We also presented a robust comparative analysis to confirm that the unaffected unilateral eyes look quite similar to normal healthy eyes and are clearly different from other keratoconic eyes. This comparative analysis benefits from the inclusion of good and representative controls from the Rotterdam Study. With a median (interquartile range) age of 53.6 (48.4–58.4) years, this control cohort significantly surpasses the common age of KC onset. Notably, a recent analysis of the prevalence of KC in the Rotterdam Study detected one tomographically unilateral KC case (Shabani et al., 2025). This represents an intriguing finding given that the patient was 67.5 years old.

Some of the used inclusion criteria such as the final Belin‐Ambrosio D‐index (BAD‐D) were somewhat related to parameters later evaluated in the comparative analysis. This might raise concerns about circular reasoning or data leakage. However, we believe these concerns do not compromise the validity of our findings for several reasons. First, our selection process employed a comprehensive set of nine criteria, which reduced the influence of any single criterion and minimized the risk of selection bias. Second, given the inherent interrelation of tomographic parameters, some degree of correlation between inclusion and tested variables is hard to avoid. This is particularly true when selecting the most asymmetric cases, as ensuring fellow‐eye normality requires the application of several strict inclusion criteria. Third, the variables analysed in the comparative analysis offer unique insights and are not fully correlated with the inclusion criteria. For instance, cases with a normal overall BAD‐D score might have a few elevated BAD‐D subcomponents and vice versa. Lastly, the primary aim of our study was confirmatory: To demonstrate the existence of tomographically unilateral KC cases with tomographically normal fellow eyes. This objective can still be robustly achieved despite some correlation between inclusion criteria and the variables analysed in the comparative analysis.

One of the criteria used to determine tomographic unilaterality was the absence of a *K*_max_ (maximum keratometry) increase exceeding 1 dioptre in the seemingly healthy eye during follow‐up (Table 1). While this cut‐off is widely utilized in studies and legal contexts to define progression, it may be suboptimal in eyes with lower baseline *K*_max_ values (Gustafsson et al., 2020). Nevertheless, the application of additional selection criteria ensured that the risk of misclassifying progressive eyes as tomographically normal was minimal. This is further supported by the comparative analysis results, which confirmed that UL‐fellow eyes have Pentacam readings that are comparable to the matched, healthy control eyes.

It might be argued that a follow‐up of 36 months is still insufficient to rule out the possibility of future ectasia in the UL‐fellow eyes. However, our case series included interesting unilateral patients whose unaffected fellow eyes remained stable for considerably longer times (up to 12.6 years). These eyes are very unlikely to change or deteriorate by longer follow‐up. Furthermore, seven (39%) of the presented tomographically unilateral cases were older than 35 years at the last follow‐up, which indicates that their unaffected corneas are considerably less prone to developing KC (McMahon et al., 2006).

Our study focused on stable tomographically unilateral KC cases. However, to definitively classify a case as genuine unilateral KC, one must completely rule out any trace of ectasia. This requires using additional imaging modalities that were not available in this case series, such as biomechanical imaging or epithelial thickness mapping. Previous reports suggested that forme fruste eyes can be distinguished from normal eyes using (combinations of) biomechanical assessment, Scheimpflug tomography and/or optical coherence tomography (Ambrósio et al., 2023; Fraenkel et al., 2021; Luz et al., 2016; Saad & Gatinel, 2010; Temstet et al., 2015; Tian et al., 2021; Zhang et al., 2015). Notably, these studies include patients with varying degrees of very asymmetric ectasia in the forme fruste group. In contrast, our case series focuses solely on the very extreme end of the KC asymmetry spectrum. In selecting these cases, we took into consideration the normality of the anterior surface attributes, posterior surface attributes, pachymetry, pachymetry distribution and stability across follow‐up. Isolated case reports have also suggested that unilateral KC cases with completely normal biomechanics and tomography may exist (Di Felici et al., 2023; Saad et al., 2022).

## 5 CONCLUSION

In conclusion, the data presented in this study suggest that some patients might have stable forms of tomographically unilateral KC. Given the identical genetic predisposition, these disease forms represent an environmentally driven phenotypic polymorphism. As such, they can be exploited to learn more about the pathogenesis of KC. In particular, a comparative multi‐omics approach that contrasts the affected and unaffected eye pairs can potentially be applied to identify relevant KC biomarkers and mechanisms. Moreover, comparing the polygenic risk scores of unilateral KC patients with bilateral KC patients may unveil interesting similarities or differences in the genetic drivers affecting these two groups.

## Supporting information


Table S1

Table S2
