# Supplementary material for: Extremely asymmetric ectasia: Tomographically unilateral keratoconus
Source: Acta Ophthalmol. 2025 Feb 7;103(5):530–8. doi: 10.1111/aos.17456 (PMC12235680; doi:10.1111/aos.17456)
Supplement: Supplementary file 1 — Table S1 [file AOS-103-530-s002.docx]

| **Supporting Table S1.** Study Groups | |
| --- | --- |
| **UL-Fellow** | healthy eyes of tomographically unilateral cases |
| **UL-KC** | keratoconic eyes of tomographically unilateral cases |
| **Fellow-Matched-KC** | bilateral keratoconic eyes that were matched the UL-Fellow group |
| **UL-Matched-KC** | bilateral keratoconic eyes that were matched to the UL-KC group |
| **Controls** | healthy eyes that were matched to the UL-Fellow group |
