# Supplementary material for: Extremely asymmetric ectasia: Tomographically unilateral keratoconus
Source: Acta Ophthalmol. 2025 Feb 7;103(5):530–8. doi: 10.1111/aos.17456 (PMC12235680; doi:10.1111/aos.17456)
Supplement: Supplementary file 2 — Table S2 [file AOS-103-530-s001.docx]

| **Supporting Table S2.** Mean ± Standard Deviation for the 25 Analyzed Pentacam Parameters | | | | | |
| --- | --- | --- | --- | --- | --- |
|  | **Controls**  **(N=900)** | **UL-Fellow**  **(N=18)** | **UL-KC**  **(N=16)** | **UL-Matched-KC**  **(N=48)** | **Fellow-Matched-KC**  **(N=54)** |
| **BAD-Df** | -0.221 ± 1.190 | 0.118 ± 1.009 | 8.734 ± 4.003 | 7.887 ± 5.526 | 8.686 ± 7.790 |
| **BAD-Db** | 0.359 ± 1.057 | -0.034 ± 0.665 | 7.428 ± 3.131 | 6.624 ± 4.311 | 6.991 ± 5.762 |
| **BAD-Dp** | 0.158 ± 0.966 | 0.366 ± 0.725 | 6.490 ± 3.864 | 7.755 ± 5.023 | 7.844 ± 5.233 |
| **IHA** | 6.497 ± 5.801 | 6.539 ± 4.950 | 32.206 ± 16.658 | 29.683 ± 29.039 | 26.552 ± 20.597 |
| **IHD** | 0.014 ± 0.009 | 0.011 ± 0.006 | 0.113 ± 0.046 | 0.100 ± 0.061 | 0.102 ± 0.074 |
| **IS-Value** | -0.182 ± 0.875 | 0.302 ± 0.665 | 6.152 ± 2.915 | 5.490 ± 3.624 | 5.247 ± 3.828 |
| **ISV** | 17.698 ± 7.364 | 17.778 ± 5.714 | 80.438 ± 29.635 | 77.583 ± 34.381 | 73.741 ± 42.730 |
| **IVA** | 0.151 ± 0.080 | 0.116 ± 0.062 | 0.951 ± 0.419 | 0.859 ± 0.446 | 0.804 ± 0.514 |
| **ART-avg** | 625.026 ± 118.470 | 569.889 ± 67.114 | 286.063 ± 75.766 | 259.354 ± 102.609 | 263.167 ± 117.997 |
| **ART-min** | 911.512 ± 240.182 | 837.389 ± 149.418 | 431.750 ± 215.168 | 380.312 ± 203.963 | 391.241 ± 203.194 |
| **K2BD** | -6.385 ± 0.277 | -6.344 ± 0.248 | -7.163 ± 0.599 | -7.129 ± 0.778 | -7.248 ± 0.882 |
| **K2FD** | 43.593 ± 1.551 | 43.400 ± 1.273 | 47.306 ± 2.647 | 47.985 ± 3.776 | 48.483 ± 4.270 |
| **KmBmm** | -6.236 ± 0.251 | -6.144 ± 0.215 | -6.750 ± 0.505 | -6.733 ± 0.679 | -6.898 ± 0.817 |
| **KmFD** | 43.147 ± 1.471 | 42.739 ± 1.184 | 45.638 ± 2.147 | 46.323 ± 3.351 | 47.085 ± 3.993 |
| **ARC** | 7.852 ± 0.272 | 7.891 ± 0.227 | 6.879 ± 0.474 | 6.891 ± 0.506 | 6.796 ± 0.629 |
| **PRC** | 6.355 ± 0.258 | 6.422 ± 0.194 | 5.149 ± 0.534 | 5.177 ± 0.558 | 5.131 ± 0.647 |
| **RMS (HOA) CB** | 0.189 ± 0.045 | 0.107 ± 0.016 | 0.284 ± 0.094 | 0.728 ± 0.309 | 0.705 ± 0.386 |
| **RMS (HOA) CF** | 0.569 ± 0.276 | 0.260 ± 0.070 | 1.128 ± 0.457 | 2.496 ± 1.230 | 2.497 ± 1.561 |
| **RMS (HOA) C** | 0.605 ± 0.312 | 0.242 ± 0.059 | 0.908 ± 0.393 | 2.184 ± 1.101 | 2.217 ± 1.421 |
| **Z(3,-1) CB** | -0.011 ± 0.059 | 0.011 ± 0.028 | 0.204 ± 0.092 | 0.527 ± 0.345 | 0.504 ± 0.378 |
| **Z(3,-1) CF** | 0.087 ± 0.294 | -0.054 ± 0.129 | -0.914 ± 0.422 | -1.949 ± 1.284 | -1.935 ± 1.400 |
| **Z(3,-1) C** | 0.094 ± 0.315 | -0.042 ± 0.117 | -0.746 ± 0.358 | -1.690 ± 1.152 | -1.704 ± 1.273 |
| **Z(3,1) CB** | 0.005 ± 0.040 | 0.000 ± 0.022 | -0.010 ± 0.079 | -0.083 ± 0.271 | -0.034 ± 0.274 |
| **Z(3,1) CF** | -0.012 ± 0.177 | -0.035 ± 0.077 | 0.056 ± 0.299 | 0.224 ± 0.967 | 0.212 ± 1.010 |
| **Z(3,1) C** | -0.009 ± 0.187 | -0.036 ± 0.065 | 0.050 ± 0.237 | 0.176 ± 0.843 | 0.206 ± 0.894 |
| Values were taken from the last reliable Pentacam scan (taken before corneal operations, if operated). The variables here are presented in their original (unscaled) format. ARC, anterior radius of curvature in the 3-mm zone centered around the thinnest pachymetry; ART-avg, average Ambrosio Relational Thickness; ART-min, minimum Ambrosio Relational Thickness; BAD-Db, standardized back elevation difference between the best-fit sphere and the enhanced reference surface; BAD-Df, standardized front elevation difference between the best-fit sphere and the enhanced reference surface; BAD-DP, C, total cornea; CB, corneal back surface; CF, corneal front surface; standardized average pachymetric progression index; IHA, Index of Height Asymmetry (microns); IHD, Index of Height Difference (microns); ISV, Index of Surface Variance; IS-Value, Inferior-Superior Value (diopters); IVA, Index of Vertical Asymmetry (millimeters); K2BD, mean keratometry in the steepest meridian of the cornea's back (diopters); K2FD, mean keratometry in the steepest meridian of the cornea's front (diopters); KmBmm, mean back surface keratometry (millimeters); KmFD, mean front surface keratometry (diopters); PRC, posterior radius of curvature in the 3-mm zone centered around the thinnest pachymetry; RMS (HOA), root mean square of high order aberrations (microns); Z(3,1), horizontal coma (microns); Z(3,-1), vertical coma (microns). | | | | | |
